# Implications of Using Different Methods to Characterise Anticoagulant Control in Patients with Second Generation Mechanical Heart Valve Prostheses

**DOI:** 10.1371/journal.pone.0098323

**Published:** 2014-07-02

**Authors:** Francesca Fiorentino, Chris A. Rogers, Alan J. Bryan, Gianni D. Angelini, Barnaby C. Reeves

**Affiliations:** 1 Bristol Heart Institute, University of Bristol, Bristol Royal Infirmary, Bristol, United Kingdom; 2 National Heart & Lung Institute, Imperial College London, Hammersmith Hospital, London, United Kingdom; Maastricht University Medical Center, Netherlands

## Abstract

**Objective:**

Characterisation of anticoagulant control is fundamental to investigations of its association with clinical outcome. Anticoagulant control depends on several factors. This paper aims to illustrate the implications of different methods for measuring and analysing anticoagulant control in patients with second generation mechanical heart valve prostheses.

**Methods:**

International normalised ratio (INR) data collected during the 10-year follow-up of a randomised controlled trial were analysed. We considered the influence of: 3 different target INR ranges; anticoagulant control expressed as the proportion of INR readings (PoR) vs. anticoagulant control follow-up time (PoT); 3 ways of describing the profile of anticoagulant control over time.

**Results:**

Different target INR ranges dramatically influenced derived measures of anticoagulant control; the PoT within the target range varied from 88% for the widest to 28% for narrowest range. Overall distributions of PoR and PoT observations were similar but differed by up to ±20% for individuals; PoT exceeded PoR when control was good but was less than PoR when control was poor. Classifying PoT outside the target range showed that widely varying combinations of PoT too high and too low are possible across individuals.

**Conclusions:**

Researchers' choices about methods for measuring and quantifying anticoagulant control markedly influence the values derived from INR readings. The use of different methods across studies makes it difficult or impossible to compare findings and to establish an evidence base for clinical practice. Methods for quantifying anticoagulant control should be standardised.

## Introduction

Patients with mechanical heart valve prostheses are maintained on lifelong anticoagulation to reduce the risks of systemic thromboembolism and valve thrombosis. The international normalised ratio (INR) is widely used to provide a reliable standardised measure of anticoagulant control [1]. Anticoagulation also poses an iatrogenic risk to health from haemorrhage and it is important to maintain a level of anticoagulation that minimises the combined thromboembolic and haemorrhagic risks.

Interest in optimising the level of anticoagulant control has led to research studies of the association between anticoagulant control and clinical outcome [2]–[10]. Characterising anticoagulant control is fundamental to such studies and depends on several factors:

- Definition of target INR range: In a clinical population, it is not feasible to analyse anticoagulant control as a continuously varying quantity (i.e. INR) over time. Therefore, researchers have tended to derive aggregate measures of anticoagulant control, such as the proportion of follow-up time during which a patient's INR was within a specified target range. This percentage depends critically on the target range adopted.
- The method used to measure anticoagulant control: Azar et al. [11] described several alternatives that have been used by researchers to translate INR readings at discrete time points into a measure of anticoagulant control.
- The method of describing the profile of anticoagulant control over time: many studies have described anticoagulant control by deriving some measure of the extent to which a patient's INR was within a specified target range during follow-up. However, the consequences of being outside the target range may differ depending on (a) the proportion of time during which a patient's INR was too high or too low during follow-up and (b) the extent to which the INR diverged from the target range. Two patients with the same proportion of follow-up time within the target range could have very different profiles of time out of control, e.g. one mainly high, the other mainly low, potentially with different clinical consequences; similarly, two patients with the same proportion of follow-up within the target range could have INR levels which diverged from the target range to a greater or less extent when outside the target range.
- The method of analysis: Anticoagulant control can be characterised across all available follow-up, or as a ‘time varying’ measure (multiple measures of anticoagulant control determined for different periods of follow-up, e.g. 6 monthly or annually).

Any attempt to characterise anticoagulation control involves a compromise. Measurements of anticoagulation using INR are subject to inter-individual biological variation in the plasma levels of coagulation factors (for a given INR) and to imprecision in INR measurement in the laboratory. INR can also vary within individual patients over time due to fluctuations in dietary vitamin K, drug interactions or poor compliance.

The aim of this paper is to illustrate how decisions about factors A-D can have important implications for the characterisation of anticoagulant control and, hence, for studies that aim to measure anticoagulant control or investigate associations between anticoagulant control and outcome. We also report a new categorical ‘profile’ measure of anticoagulant control to characterise the predominant nature of the deviation when a patient is not well controlled.

## Methods

Data from an existing randomised controlled trial (RCT) comparing two second generation mechanical bileaflet heart valves, St Jude Medical and Carbomedics, were used to illustrate the consequences of different decisions about factors A, B and C. Details of the methods of this study, and 5 and 10 year follow-up with respect to mortality and valve-related events (the key outcomes in the RCT), have been reported previously [12], [13]. No difference in outcome between valves was observed [12], [13] and the analyses reported in this paper treat patients from both arms of the trial as a single cohort. The trial was approved by a UK National Health Service Research Ethics Committee. Anonymised data were analysed for this paper.

The cohort was divided into two groups; (a) participants who had an aortic valve replacement only (AVR) and (b) participants who had a mitral valve replacement with or without an aortic valve replacement (MVR).

### Description of follow-up methods

Follow-up information was obtained by an annual questionnaire to survivors (by telephone), requesting participants to provide the last 10 INR readings for the year of follow-up (from their anticoagulant history booklet filled out by the physician), and details of any thromboembolic or bleeding events requiring hospitalisation. Participants who did not send back their questionnaires were sent a reminder and were then telephoned. Clinical events were clarified by contacting the participant's family physician or hospital cardiologist when there was uncertainty about the details of the event.

When INR readings were unstable or out-of-range, it is likely that more frequent measurements were taken until the INR was back in-range. There may also have been periods when anticoagulation might have been suspended or reversed for a surgical procedure or serious bleeding. Even in such cases the last 10 INR readings available from the patient diary were used, to avoid restricting the variability of anticoagulation control when patients' ongoing management or other circumstances may have caused the INR to become unstable.

### Factor A: Definitions of target INR ranges

The RCT on which analyses in this paper are based was carried out between 1992 and 2004. During this time, three target INR ranges were being recommended in guidelines and are likely to have directed anticoagulation for trial participants:

- 2.0 to 4.0 [3];
- 2.5 to 3.5 [14];
- 2.5 to 3.0 for the AVR group and 3.0 to 3.5 for the MVR group [15].

Since the trial concluded, more recent recommendations have been made [16] and usual practice has tended to use lower INR ranges, largely based on observational evidence that lower INR values are safe. We have nevertheless carried out the analyses with the above INR ranges because they are contemporary for the period of the trial and allow us to illustrate the consequences of adopting different ranges.

### Factor B: Measures of anticoagulant control

We investigated two ways of measuring anticoagulant control, namely the proportion of follow-up INR readings [3] and the proportion of follow-up time, when the INR was within the specified target range, too low or too high. When calculating the proportion of follow-up time, the INR level was interpolated on a daily basis from the dates on which INR readings were available [11]. Other measures exist [11] but were not considered here.

The first method expresses the proportion of INR readings within the defined target range as a proportion of all INR readings during follow-up, providing ≥10 INR readings are available. This measure is abbreviated here to ‘percentage of readings’ in control, too low or too high (PoR_in control_, PoR_too low_. PoR_too high_). It has been reported to give the lowest estimates of anticoagulant control [11]. The method takes no direct account of time under observation, unless INR readings are assumed to be available at constant intervals for all patients under observation for the entire duration of follow-up.

The second method is based on interpolating anticoagulant control on a day-to-day basis, assuming that the INR level changes linearly between readings. Anticoagulant control is expressed as a ‘percentage of time’ (PoT_in control_, PoT_too low_. PoT_too high_). If gaps in time between readings are longer than 56 days, these periods of time do not contribute to PoT_in control_ for individual patients. (There were gaps of this kind during follow-up for our dataset because the last ten sequential INR readings provided by patients often did not cover a full year; **Figure S1**.)

### Factor C: Describing the profile of anticoagulant control over time

As well as calculating the PoT_in control_ for different target INR ranges, we divided the *time out of control* into the PoT when the INR was too low (PoT_too low_) and too high (PoT_too high_). PoT_in control_, PoT_too high_ and PoT_too low_ are correlated because they must sum to the total eligible follow-up time.

In order to describe the ***profile*** of anticoagulant control for individual patients, we created four mutually exclusive categories based on PoT_in control_, PoT_too high_ and PoT_too low_:

- Good: PoT_in control_≥67%
- Fair: (PoT_in control_<33%) AND (PoT_too low_≥33%) AND (PoT_too high_≥33%)
- Too high: (PoT_too high_≥33%) AND (PoT_too low_<33%)
- Too low: (PoT_too low_≥33%) AND (PoT_too high_<33%)

Fair control describes INR outside the target range most of the time but approximately centred on the target range, with roughly equal PoT_too low_ and PoT_too high_. ‘Too high’ and ‘too low’ represent INR predominantly high or low, respectively, when out of range.

We also divided the distribution of observations of PoT_in control_ in two ways, to create two ordinal measures of anticoagulant control, each with four levels:

- four groups with equal numbers of observations (i.e. quartiles of the distribution);
- four groups of equal width defined according to the PoT_in control_ (i.e. 0–24%, 25–49%, 50–74%, 75–100%).

We created ordinal variables for the PoT_too high_ and PoT_too low_ in the same way. Like the profile measure, these ordinal measures of PoT_in control_, PoT_too high_ and PoT_too low_ collectively characterise the predominant deviation in an individual when the INR is not in control but require three variables to be inspected rather than one. (Strictly, only two of the three variables need to be examined because the third can be derived from the other two.)

We calculated all measures of anticoagulant control both for the entire duration of follow-up for a participant and annually for full or part-years of follow-up. The means/medians of the distributions for overall and annual measures will be similar if anticoagulant control does not change systematically over time. However, the distribution of the overall measure will inevitably be less dispersed because outlying annual observations will be smoothed when combined over all years of follow-up.

### Statistical analysis

Cumulative frequency distributions of PoR_in control_ and PoT_in control_ were generated and summarised graphically for different target INR ranges. Discrepancies between overall measures of PoR_in control_ and PoT_in control_ were illustrated by Bland-Altman limits of agreement plots [17]. Changes in categorisation of anticoagulant control over successive years were summarised as cross tabulations. Our aim was purely descriptive and we did not use statistical hypothesis tests except when examining the relationships between differences and averages in Bland-Altman plots. All analyses were carried out using STATA v12.1 (Stata Corporation, Texas).

## Results

### Study population

Details of the study population have been described elsewhere [12], [13]. Participants (n = 485) were recruited from 1992 to 1996; 69 who provided <10 INR readings are excluded from the analyses. INR data for the remaining 416 were available at latest up to 31 December 2004 when data collection stopped. The total observation time for these 416 participants was 3,499 patient years.

Patients with insufficient INR data were more likely to have died during follow-up (57/69, 82.6%; 32/42 in the AVR group and 25/27 in the MVR group) than patients with adequate INR data (108/416, 26.0%; 49/247 in the AVR group and 59/169 in the MVR group). Similar percentages of patients in AVR and MVR groups had insufficient INR data for analysis (AVR, 42/289, 14.5%; MVR, 27/196, 13.8%). Only 16 of 108 deaths during follow-up were valve related.

Of 416 participants who had ≥10 INR readings during follow-up, there were 247 in the AVR group and 169 in the MVR group. A total of 27,383 unique, valid INR readings were documented in these patients; 110 did not have dates and were excluded. A total of 23,946 INR readings (87.8% of 27,273) occurred within 56 days after the preceding measurement. The median gap between measurements was 21 days (inter-quartile range 13 to 30 days; see **Figure S1**) with a distribution that reflected scheduling of measurements at weekly intervals; the distribution did not differ by AVR/MVR group (**Figure S1, b&c**). The other 3,327 INR readings which occurred more than 56 days after the preceding measurement caused gaps in the INR record; these gaps arose mainly (65% of 3,327) because of periods of time between sequences of 10 readings recorded for different years of follow-up.

The median duration of follow-up was 9.0 years and the median number of INR readings per participant was 77 (range 10 to 110). The cumulative frequency distributions of all INR readings were very similar for the AVR and MVR groups; medians, 75th and 99th centiles were 3.0/3.5/5.6 and 3.1/3.6/5.8 respectively (**Figure S2**).

### A. Definition of target INR range

**Figure 1:**
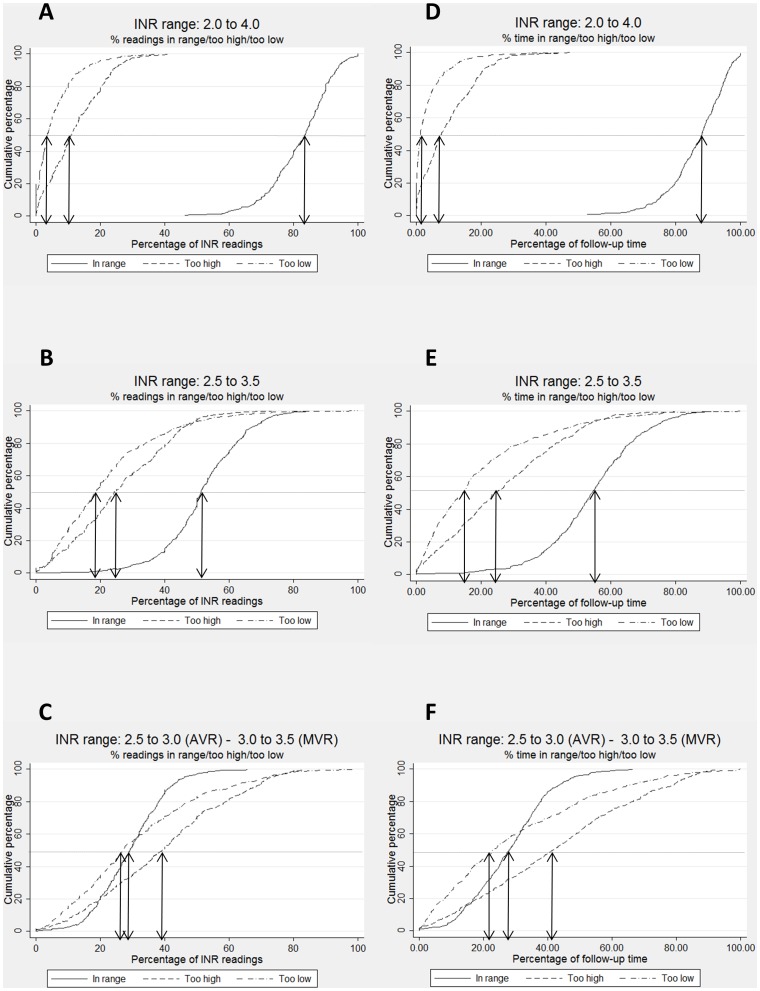
Cumulative percentages of the proportion of readings (panels A, B and C) and follow-up time (panels D, E and F) in range, too high and too low. Results are shown for the three different target INR ranges investigated: I - 2.0 to 4.0 (panels A and D); II - 2.5 to 3.5 (panels B and E); III - 2.5 to 3.0 for the AVR and 3.0 to 3.5 for the MVR groups (panels C and F).

**Figure 1** shows cumulative frequency distributions for PoR (left column) and PoT (right column), for PoR and PoT_in control_ (solid lines), PoR and PoT_too high_ (dashed lines) and PoR and PoT_too low_ (dotted-dashed lines) for the three target INR ranges that were considered. All measures were calculated across the whole period of follow-up for individual participants. The better the anticoagulant control, the further to the right the solid line should be, and the further to the left of the panel the dashed, and dotted-dashed lines, should be. As anticoagulant control deteriorates, the solid line will move to the left and the other lines to the right. The shallower the gradient of the lines, the greater is the variability in anticoagulant control between individuals.

Not surprisingly, anticoagulant control appears best with the widest target INR range I, worst with the most specific range III and intermediate with range II. The magnitude of the differences in the distributions for different target INR ranges is, perhaps, less expected. For example, the median PoT_in control_ for target INR range I is 88%, 54% for range II, but only 28% for range III. For all three INR ranges, participants were slightly more likely to have an INR that was too high rather than too low, i.e. the dotted-dashed lines lie consistently to the left of the dashed lines in all panels of **Figure 1**.

### B. Comparison of PoR and PoT measures of anticoagulant control

The cumulative frequency distributions in **Figure 1** for measures based on PoR and PoT look remarkably similar. Medians for the PoR_in control_ and PoT_in control_ for the three target ranges were: 84% and 88% (panels A and D); 51% and 54% (panels B and E); 29% and 28% (panels C and F). Medians for POR_too high_ and POT_too high_ (11% and 8% for INR range I; 24% and 24% for INR range II; 39% and 42% for INR range III), and POR_too low_ and POT_too low_ (3% and 1% for INR range I; 19% and 14% for INR range II; 27% and 23% for INR range III) were also similar. However, the figures show cumulative distributions across all participants, so do not represent discrepancies between PoR and PoT measures for individuals.

**Figure 2:**
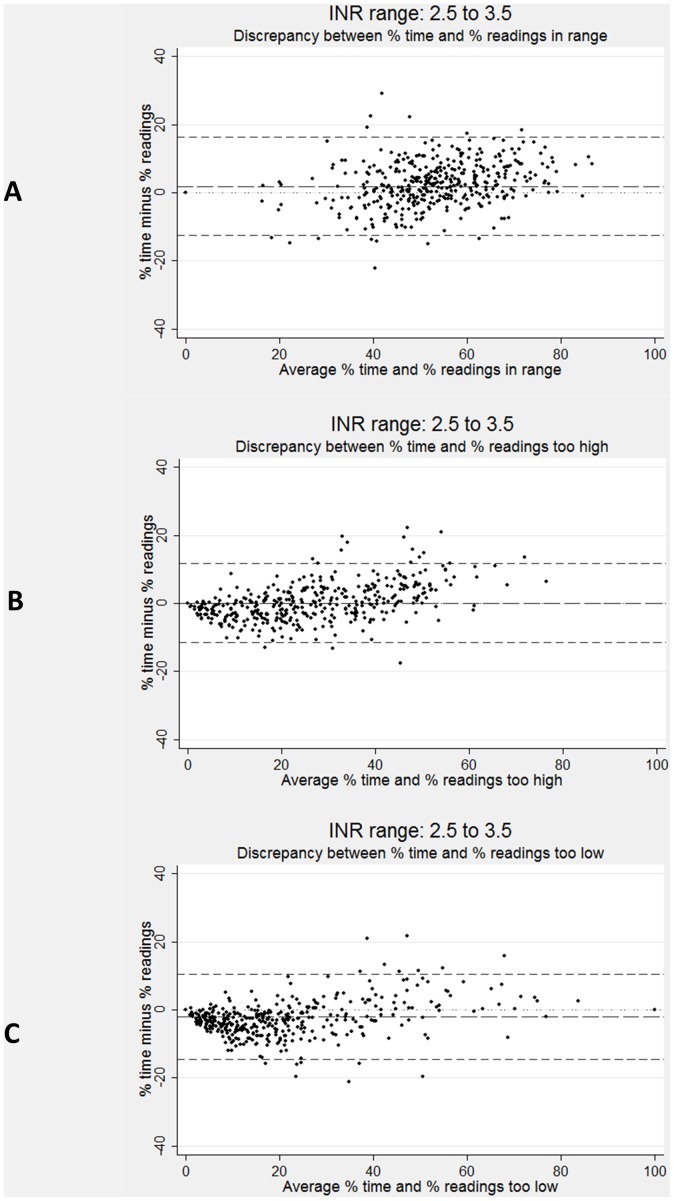
Limits of agreement plots comparing proportion of readings and proportion of time in range, too high and too low for target INR range II (2.5 to 3.5). The long-dashed line represents the difference between the % of readings and the % of time; the short-dashed line represents the 95% limits of agreement.

Discrepancies between PoR and PoT measures for individuals are shown as Bland-Altman plots in **Figure 2** for the target INR range II, for PoR and PoT_in control_, PoR and PoT_too high_ and PoR and PoT_too low_. These graphs highlight that discrepancies for individuals can range from −20% to +20%; the standard deviations of the differences between PoT and PoR were about 7% for each pair of measurements. Note that the graphs show PoR and PoT values when calculated across the whole period of follow-up for individual participants; the discrepancies were substantially larger between annual measures of PoR and PoT values, because values were more dispersed. **Figure S3** shows discrepancies as Bland-Altman plots for the target INR ranges I and III.

There were statistically significant associations between the difference and the average for all three plots, i.e. regression lines fitted to the data had a positive gradient (all p<0.001). For PoR and PoT_in control_, the fitted PoT value was greater than PoR (i.e. positive difference, data point towards upper right quadrant of panel in **Figure 2A**) above an average of 41% and smaller below (i.e. negative difference, data point towards lower left quadrant). For PoR and PoT_too high_ and_too low_, the fitted PoT was also higher than PoR for the majority of the range, above 24% and 40% respectively.

### C. Characterising the profile of anticoagulant control

**Figure 3:**
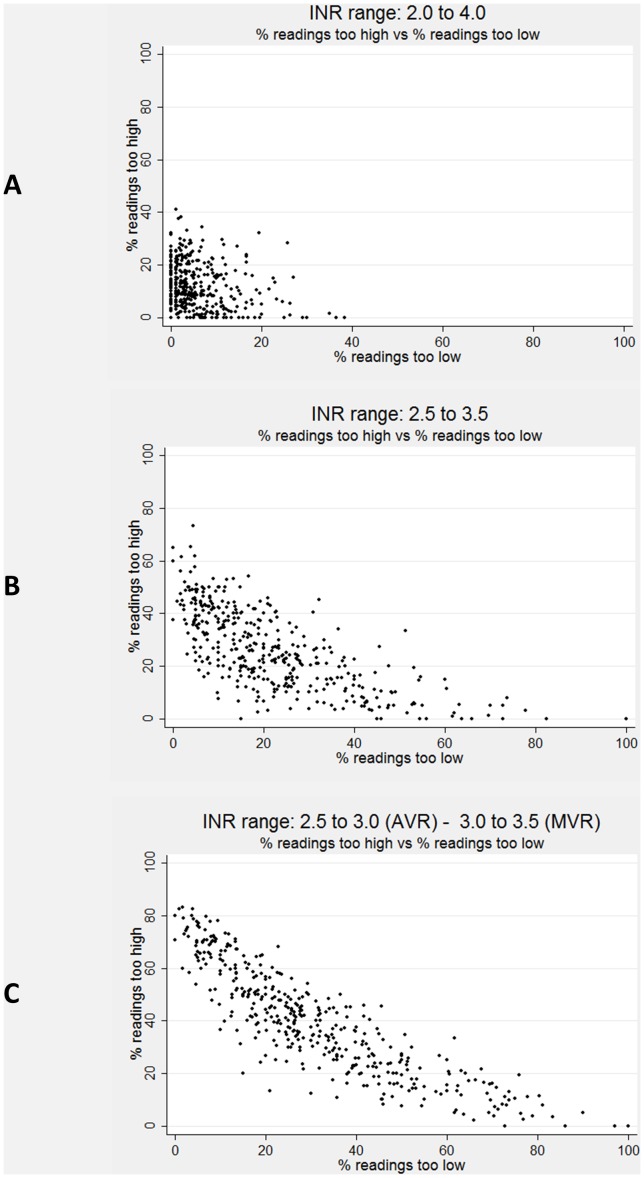
Plot of proportion of time too high and too low for target INR range I (2.0 to 4.0), INR range II (2.5 to 3.5) and INR range III (2.5 to 3.0 for the AVR and 3.0 to 3.5 for the MVR groups).

The potential importance of considering whether the INR level was too high or too low when out of range is illustrated in **Figure 3**, which plots PoT_too high_ against PoT_too low_ for the three target INR ranges considered. The distance of points furthest from the origin depends on the median PoT_too high_ and PoT_too low_, and hence the target INR range. For example, with a wide target range, when the medians for PoT_too high_ and PoT_too low_ are low, the extreme values on each axis, and combinations of PoT_too high_ and PoT_too low_, will be constrained to be closer to the origin (**Figure 3A**). Within the boundary formed by the most extreme values, almost any combination of PoT_too high_ and PoT_too low_ appears to be possible (**Figure 3C**).

A final issue concerns the period over which a measure of anticoagulant control is aggregated. A decision about this should depend on the precise hypothesis being investigated. Here, we seek only to demonstrate that measures of anticoagulant control vary over time. Therefore, aggregation over an increasing duration of time will tend to obscure this variation. **Table 1** illustrates the extent to which annual measures of anticoagulant control change from one year to the next, for the INR range II. The cells in the table represent percentages of participants with anticoagulant control classified as tabulated in consecutive years. (Participants contribute multiple observations to this table because the aggregate measure of anticoagulation control for year_n_ is compared to aggregate measures of anticoagulation control for both year_n−1_ and year_n+1_.)

**Table 1:** Comparison of the classification of annual observations of anticoagulant control for INR range II for the same patients in consecutive years; (a) the proportion of follow-up time (PoT)_in control_, (b) profile measure of anticoagulant control.

(a) PoT_in control_					
	Year_(i+1)_				
Year_(i)_	0–24%	25–49%	50–74%	75–100%	Total
0–24%	2.6	3.4	5.1	1.4	12.5
25–49%	4.1	6.9	9.8	4.9	25.7
50–74%	4.4	9.2	19.2	8.6	41.3
75–100%	1.4	4.2	9.1	5.8	20.6
Total	12.5	23.6	43.2	20.7	100.0

(b) Profile measure of anticoagulant control					
	Year_(i+1)_				
Year_(i)_	Too low	Good	Fair	Too high	Total
Too low	7.6	5.5	3.5	1.9	18.6
Good	4.4	11.4	5.1	8.7	29.7
Fair	3.4	5.0	6.8	5.6	20.7
Too high	2.1	7.7	5.4	15.9	31.0
Total	17.4	29.7	20.8	32.1	100.0

Changes between all categories were observed and only a minority of pairs (34.5%, i.e. 2.6%+6.9%+19.2%+5.8%) had PoT_in control_ classified in the same ordinal category in successive years (**Table 1a**). Classification of anticoagulant control in successive years is not markedly more similar when the profile measure of anticoagulant control is used (which takes into account PoT_too high_ and PoT_too low_); 42% of individuals had anticoagulant control classified in the same category in successive years (**Table 1b**). Agreement is somewhat better for PoT_too high_ (52%) and PoT_too low_ (66%) (**Tables S1a** and **S1b**). The proportion of measures of anticoagulant control classified in the same way in successive years depends on the target INR range chosen. (Tables for different target INR ranges are available from the authors.)

## Discussion

Our findings show that choices about the target INR range and the methods used for aggregating data about INR levels and quantifying anticoagulant control all markedly influence the derived values of anticoagulant control. Calculating aggregated measures of anticoagulant control over long durations of follow-up will inevitably obscure any association between anticoagulant control and outcome over short periods of time. This is not an academic matter since it is clear that researchers make different choices about these factors [2]–[10].

Our findings arise from INR data collected in the context of a randomised trial, which did not constrain usual management of INR during follow-up and where the INR measurements did not constitute an outcome. It is important to acknowledge that the INR range to be analysed in a study should depend on the purpose of the analysis. If the purpose is to audit the competence of anticoagulation clinics (an administrative requirement), a narrower range centred on the prescribed target INR for a particular indication is appropriate. If the purpose is to relate INR control to outcome measurements after valve replacement, such as thromboembolism, valve thrombosis, major bleeding and survival, choosing a wider INR range may be more useful.

### Strengths and limitations

Our dataset was relatively small but still included >27,000 individual INR readings and almost 3,500 person years of follow-up. Collection of the data in the context of a RCT is an important strength of the dataset. Participants in the trial had only one of two types of prosthesis. Although the type of heart valve may interact with the association between anticoagulant control and clinical outcome [4], this does not limit the applicability of our observations about *measuring* anticoagulant control. The distribution of INR readings in this study was very similar to that of Cannegieter and colleagues [4] and slightly less dispersed than that of Butchart and colleagues [3] In so much as self-monitoring regimens for anticoagulant control achieve better control [6]–[8], PoT_in control_ with self-monitoring might be expected to be less variable over time than observed with our data. However, since INR readings still vary over time with self-monitoring, these regimens would not change our findings with respect to different target ranges and different methods of measuring anticoagulant control.

The INR ranges that we considered relate to guidelines that were contemporary with the data collection. Current guidelines tend to recommend target INRs, e.g. 2.5 for AVR and 3.0 for MVR, rather than target ranges, in order to avoid the extremes of the ranges being considered equally acceptable by anticoagulation clinics. However, any analysis of the adequacy of anticoagulation control over time (as opposed to the INR target in an anticoagulation clinic) still needs to set a range within which control is considered satisfactory. To this extent, our findings are as applicable now as they were during follow-up to the trial.

There were some gaps in the INR record for participants. Most of these arose because of the way the data were collected and do not affect the representativeness of the dataset. A small proportion of INR readings (about 4%) were >56 days after the preceding reading and within the sequence of the “last 10 measurements” collected at the annual follow-up. The distribution of time between readings beyond 56 days formed a smooth tail and we have no reason to suspect that omitting these periods of time could have introduced bias.

### Proportion of time versus proportion of readings within the target range

Azar and colleagues, citing Duxbury, pointed out that PoR_in control_ may be biased if the number of readings per patient varies and is correlated with anticoagulant control [9]. They compared different methods for quantifying anticoagulant control, highlighting that it is PoT_in control_ rather than PoR_in control_ that is important. We completely concur with this view and recommend that interpolating the INR level between readings should always be used in future.

Azar and colleagues, based on a single target range (2.8 to 4.8) found that PoR_in control_ was consistently, and considerably, less than PoT_in control_ (63% and 77% overall, respectively). In contrast, we found that medians for PoR and PoT_in control_ were similar and that neither was consistently larger. The difference between medians was greatest (PoR_in control_ being smaller) for the widest target range investigated (2.0 to 4.0) and we suspect that the direction of the difference depends on the target INR range used. We believe that, as the PoT_in control_ decreases, the difference becomes smaller and reverses (PoR_in control_ being larger) for very ‘strict’ target INR ranges; this view is supported by positive gradients for regression lines (not shown) fitted to the scatter plots in **Figure 2**.

### Describing the proportion of time outside the target range

Previous researchers have focused primarily on the PoT_in control_ or PoR_in control_ for a specified target range [2], [3], [5], [6], [8], [11]. However, the PoR or PoT_too high/too low_ are also likely to be important because different clinical events are associated with high and low INR levels and any target INR range represents an attempt to balance the competing risks of thromboembolic and bleeding complications.

Although follow-up time can be divided into PoT_in control_, PoT_too high_ and PoT_too low_, only two can be modelled as continuous variables at any one time because of the degrees of freedom. Choices about how to model these variables (i.e. which pair to include, whether to include quadratic terms, etc.) introduce a further source of variation. These complexities led us to develop the profile measure. Because observations are classified into mutually exclusive categories, each category can be modelled relative to ‘good’ control, in principle allowing the balance between thromboembolic and bleeding risks to be investigated directly. To the best of our knowledge, no similar measure has been reported before.

### Implications of different choices about measures of anticoagulant control

At best, when researchers make different choices, it becomes difficult or impossible to compare findings across studies and to establish an evidence base for clinical practice. More worryingly, in observational studies when choices vary systematically over time and are confounded with changes in clinical practice (e.g. heart valve design), it becomes difficult to distinguish whether differences in research findings are attributable to changes in practice or to different choices about measuring anticoagulant control. Other complexities of comparing clinical outcomes across studies have been described elsewhere [18].

The U-shaped function describing the risk between INR level and adverse outcome related to anticoagulant control [4], and the fact that INR target ranges are not centred on the same INR level [2], [3], [5], [11], [14], [15], makes researchers' choices about measures of anticoagulant control particularly critical. The profile measure of anticoagulant control reported here would be expected to vary considerably when target ranges are centred on different values because of the way in which PoT_too high_ and PoT_too low_ will be affected.

In RCTs, comparisons of anticoagulant control between groups are completely valid, irrespective of the choices made. However, the magnitude of differences observed, and their likely statistical significance, can still be substantially affected by the precise measures of anticoagulant control that are chosen.

### Conclusions

Researchers' choices about methods to derive measures of anticoagulant control markedly influence the values ultimately used for analysis. The use of different methods across studies makes it difficult or impossible to compare findings and to establish an evidence base for clinical practice. The obvious solution is to standardise methods for quantifying anticoagulant control, although it is less clear what standard should be adopted.

## Supporting Information

Figure S1 **Distribution of times between included readings for a) all participants, b) participants in the AVR group and c) participants in the MVR group.** Vertical lines represent the median (solid line) and the first and third quartiles (interquartile; dashed lines).
(DOCX)

Figure S2 **Cumulative frequency of INR readings in AVR and MVR groups.**
(DOCX)

Figure S3 **Limits of agreement plots comparing the proportion of readings and the proportion of time in range, too high and too low for target INR range I (2.0 to 4.0, figures a–c) and INR range III (2.5 to 3.0 for the AVR group and 3.0 to 3.5 for the MVR group, figures d–f).** The long-dashed line represents the difference between the percentage of readings and the percentage of time; the short-dashed line represents the 95% limits of agreement.
(DOCX)

Table S1 **Comparison of the classification of annual observations of anticoagulant control for INR range II for the same patients in consecutive years; (a) PoT too high, (b) PoT too low.**
(DOCX)
