# Supplementary material for: Implications of Using Different Methods to Characterise Anticoagulant Control in Patients with Second Generation Mechanical Heart Valve Prostheses
Source: PLoS One. 2014 Jul 2;9(7):e98323. doi: 10.1371/journal.pone.0098323 (PMC4079318; doi:10.1371/journal.pone.0098323)
Supplement: Figure S1 — Distribution of times between included readings for a) all participants, b) participants in the AVR group and c) participants in the MVR group. Vertical lines represent the median (solid line) and the first and third quartiles (interquartile; dashed lines). (DOCX) [file pone.0098323.s001.docx]

**Figure S1**

**a)**

**b)**

**c)**
