# Supplementary material for: Implications of Using Different Methods to Characterise Anticoagulant Control in Patients with Second Generation Mechanical Heart Valve Prostheses
Source: PLoS One. 2014 Jul 2;9(7):e98323. doi: 10.1371/journal.pone.0098323 (PMC4079318; doi:10.1371/journal.pone.0098323)
Supplement: Figure S3 — Limits of agreement plots comparing the proportion of readings and the proportion of time in range, too high and too low for target INR range I (2.0 to 4.0, figures a–c) and INR range III (2.5 to 3.0 for the AVR group and 3.0 to 3.5 for the MVR group, figures d–f). The long-dashed line represents the difference between the percentage of readings and the percentage of time; the short-dashed line represents the 95% limits of agreement. (DOCX) [file pone.0098323.s003.docx]

**Figure S3**

**a)**

**b)**

**c)**

**d)**

**e)**

**f)**
