# Supplementary material for: Implications of Using Different Methods to Characterise Anticoagulant Control in Patients with Second Generation Mechanical Heart Valve Prostheses
Source: PLoS One. 2014 Jul 2;9(7):e98323. doi: 10.1371/journal.pone.0098323 (PMC4079318; doi:10.1371/journal.pone.0098323)
Supplement: Table S1 — Comparison of the classification of annual observations of anticoagulant control for INR range II for the same patients in consecutive years; (a) PoT too high, (b) PoT too low. (DOCX) [file pone.0098323.s004.docx]

**Table S1**

|  |  | 1. PoT _too high_: Year_(i+1)_ | | | | |  |
| --- | --- | --- | --- | --- | --- | --- | --- |
|  |  | 0-24% | 25-49% | 50-74% | | 75-100% | Total |
| PoT _too high_ | 0-24% | 35.1 | 11.5 | 4.3 | | 1.5 | 52.4 |
|  | 25-49% | 9.5 | 11.4 | 4.5 | | 2.2 | 27.6 |
| Year_(i)_ | 50-74% | 3.3 | 4.6 | 4.2 | | 1.6 | 13.7 |
|  | 75-100% | 1.0 | 2.3 | 2.2 | | 0.8 | 6.3 |
|  | Total | 48.8 | 29.8 | 15.2 | | 6.2 | 100.0 |
|  |  |  | 1. PoT _too low_: Year_(i+1)_ | |  |  |  |
|  |  | 0-24% | 25-49% | 50-74% | | 75-100% | 0-24% |
| PoT _too low_ | 0-24% | 60.5 | 6.3 | 2.6 | | 1.5 | 71.0 |
|  | 25-49% | 11.8 | 2.8 | 1.8 | | 1.0 | 17.3 |
| Year_(i)_ | 50-74% | 3.2 | 1.5 | 1.2 | | 1.1 | 7.0 |
|  | 75-100% | 1.5 | 1.3 | 0.9 | | 1.0 | 4.7 |
|  | Total | 77.0 | 12.0 | 6.4 | | 4.6 | 100.0 |
